# miR‐140‐3p inhibits colorectal cancer progression and its liver metastasis by targeting BCL9 and BCL2

**DOI:** 10.1002/cam4.3840

**Published:** 2021-04-10

**Authors:** Dingsheng Liu, Chunsheng Chen, Mingming Cui, Hong Zhang

**Affiliations:** ^1^ Department of General Surgery Shengjing Hospital of China Medical University Shenyang People's Republic of China

**Keywords:** BCL2, BCL9, colorectal cancer, metastasis, miR‐140‐3p

## Abstract

Recent studies have identified microRNAs (miRNAs) as a compelling novel class of biomarker in colorectal cancer (CRC) development and metastasis. Here, we demonstrated that the level of plasma exosomal miR‐140‐3p in CRC patients was lower than that in healthy controls. The decreased miR‐140‐3p level was also observed in CRC patients with liver metastasis. The expression of miR‐140‐3p in CRC tissues were significantly lower than that in matched normal tissues. Functionally, miR‐140‐3p overexpression suppressed proliferation, migration, invasion, and β‐catenin nuclear translocation, as well as promoted apoptosis in LoVo cells, while inhibition of miR‐140‐3p reversed these cellular processes in HCT 116 cells. Notably, BCL9 and BCL2 were recognized as direct targets of miR‐140‐3p. BCL9 knockdown abrogated miR‐140‐3p inhibitor‐induced effects on HCT 116 cells with decreased proliferation, migration, and invasion. BCL2 knockdown increased apoptosis of miR‐140‐3p inhibitor‐transfected HCT 116 cells. In vivo experiments revealed that miR‐140‐3p overexpression inhibited tumor growth in LoVo xenograft model and diminished metastatic nodules in nude mice liver. Taken together, this work supports that miR‐140‐3p exerts as a tumor suppressor in CRC progression via targeting BCL9 and BCL2, and suggests miR‐140‐3p‐BCL9/BCL2 axis may be applied in miRNA‐based therapy and prognostication of CRC.

## Graphical abstract

MiR‐140‐3p inhibited colorectal cancer (CRC) cell proliferation, migration, invasion and β‐catenin nuclear translocation, as well as promoted apoptosis. MiR‐140‐3p served as a tumor suppressor in CRC via targeting BCL9 and BCL2. MiR‐140‐3p‐BCL9/BCL2 axis may be applied in miRNA‐based therapy and prognostication of CRC.

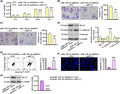

## 1 INTRODUCTION

Colorectal cancer (CRC) is the third most commonly diagnosed cancer globally, and the fourth most deadly cancer with nearly 900 000 deaths annually.^1^, ^2^ The most common cause of death in patients with CRC is the occurrence of distant metastasis. With metastasis of the liver being the most frequent site, about 25%–30% of patients diagnosed with CRC harbor liver metastases during the development of their disease.^3^ Although improvements in treatment of CRC prolonged overall survival, patients in advanced stages had poor prognosis due to metastasis and recurrence.^4^ Therefore, exploring the underlying mechanism responsible for the malignant behaviors and liver metastasis in CRC, will contribute to propose effective biomarkers and therapeutic targets for CRC.

MicroRNAs (miRNAs) are a kind of small noncoding RNAs that modulate gene expression posttranscriptionally by degrading target‐gene mRNA or inhibiting the translation of mRNA.^5^ miRNAs modulate various biological processes in many types of cancer. Furthermore, miRNAs can be encapsulated in exosomes, and exosomes containing miRNAs secrete into the circulation and transport to target cells throughout the body.^6^ Therefore, identification of exosomal miRNA levels in blood may facilitate the diagnosis of metastasis and prediction of prognosis.^7^, ^8^ A considerable amount of studies have indicated the aberrantly expressed miRNAs have a functional role in colorectal tumorigenesis and metastasis.^9^, ^10^, ^11^, ^12^, ^13^ Previous studies indicated that miR‐140‐3p is implicated in breast cancer and lung cancer, and acts as a tumor suppressor.^14^, ^15^, ^16^ Furthermore, miR‐140‐3p was found to be downregulated in CRC tissues, and was competitively combined by circSMARCC1, thereby regulating proliferation, migration, and invasion of CRC cells.^17^ It was also evidenced that miR‐140‐3p inhibits cell growth and triggers apoptosis in CRC by targeting PD‐L1 and inactivating PI3K/AKT pathway.^18^ Nevertheless, little attention has been devoted to the effects of miR‐140‐3p in CRC metastasis. Our study attempts to yield further insight into the function and detailed mechanisms of miR‐140‐3p in CRC tumorigenesis and metastasis.

BCL9 (B cell CLL/lymphoma 9), a component of Wnt/β‐catenin pathway, can form a complex with β‐catenin and activate Wnt/β‐catenin targeted‐genes to promote tumor development.^19^, ^20^ BCL9 has been proved to be upregulated in malignancies including CRC, hepatocellular carcinoma, multiple myeloma, and breast cancer.^21^, ^22^, ^23^, ^24^ For CRC, knockdown of BCL9 or blocking the interaction between BCL9 and β‐catenin inhibits tumor growth, angiogenesis, and metastasis.^21^, ^25^ BCL2, a crucial cell death regulator, locates in the outer membrane of mitochondria, where it controls the release of cytochrome c from mitochondria and suppresses the function of pro‐apoptotic genes.^26^, ^27^ High expression of BCL2 was observed in tumor tissues patients with CRC.^28^

In this work, several prediction algorithms (TargetScan, miRDB, and RNAhybrid) were employed to predict target genes of miR‐140‐3p. The prediction revealed that BCL9 and BCL2 are direct targets of miR‐140‐3p. Our study aims to explore the role of miR‐140‐3p in CRC tumorigenesis and metastasis, and whether the regulatory function of miR‑140‑3p is achieved by targeting BCL9 and BCL2.

## 2 MATERIALS AND METHODS

### 2.1 Human specimen

Human CRC blood samples (n = 70) and normal subjects (n = 30) were collected from Shengjing Hospital of China Medical University, with the consent of patients and approval by the Ethics Committee of Shengjing Hospital of China Medical University. Plasma was isolated from whole blood and stored at 80°C. Human CRC tissues (n = 24) and their matched adjacent normal tissues (n = 24) were collected from Shengjing Hospital of China Medical University, with patient consent and approval from the Ethics Committee of Shengjing Hospital of China Medical University.

### 2.2 RNA extraction from exosomes in blood

Exosome Isolation Kit (from plasma for NTA/TEM) (EZBioscience, EZB‐exo101‐L) and Exosome RNA Purification Kit (EZBioscience, EZB‐exo‐RN1) were used for isolating exosome from plasma and extracting RNA. Briefly, the plasma sample was thawed on ice. A 0.5 ml plasma sample was added into a tube and centrifuged at 3000× *g* for 10 min at 4°C. After transferring to a new tube, the supernatant was centrifuged at 10,000× *g* for 20 min at 4°C. The supernatant was transferred to a new tube and added 0.5 volume of 1× PBS. About 20% of the total volume (Total volume = plasma + PBS) Exosome Precipitation Reagent (from plasma) was added. The plasma/reagent mixture was mixed by inverting the tube and incubated at 4°C for 2 hours. After incubation, the sample was centrifuged at 10,000× *g* for 30 minuts at 4°C. The supernatant was aspirated and discarded. The tube was centrifuged for 30 seconds at 10,000× *g*, and residual supernatant was discarded. About 50 µl 1× PBS was added and resuspended the pellet. The suspension was added to a purification column, centrifuged at 4000 *g* for 5 minutes at 4°C, and the effluent was exosomes. A 500 μl of Lysis Buffer was added to exosome solution. After mixing, the sample was incubated at room temperature for 5 minutes. A 100 μl of chloroform was added to the exosome lysate, mixed by hand‐shaking, and incubated at room temperature for 3 minutes. After centrifugation at 12,000× *g*, 4°C for 2 minutes, the upper‐layer supernatant (about 300 μl) was transferred to a new RNase free 1.5 ml centrifuge tube. A 1.6 volume of 100% ethanol was added to each volume of supernatant. The centrifuge tube was inverted for 10 times to mix thoroughly, and transfer the sample to the Spin Column. After centrifugation at 4000× *g*, 4°C for 1 minute, the liquid was poured off. A 500 μl of Wash Buffer 1 was added to the column. After centrifugation at 12,000× *g*, 4°C for 1 minute, the liquid was poured off. A 500 μl of Wash Buffer 2 was added and repeated the process above. After centrifugation at 12,000× *g*, 4°C for 1 minute, the column was transferred to an RNase free 1.5 ml centrifuge tube, and kept in the air for 2 minutes. A 30 μl of Elution Buffer was added to the center of the column and incubated at room temperature for 2 minutes. After centrifugation at 12,000× *g*, 4°C for 1 min, the effluent was purified RNA.

### 2.3 Cell culture

Human CRC LoVo and HCT 116 cell lines were purchased from Procell Life Science and Technology Co., Ltd (Wuhan, China). LoVo cells were cultured in Ham's F‐12K medium (Procell Life, PM150910) supplemented with 10% of fetal bovine serum (FBS, 04‐011‐1A, BI, Israel). HCT 116 cells were cultured in McCoy's 5A medium (Procell Life, PM150710) containing 10% of FBS. Cells were maintained in a humidified incubator (37°C, 5% CO_2_).

### 2.4 Cell transfection

LoVo cells were transfected with miR‐140‐3p mimic or negative control (NC) mimic. HCT 116 cells were transfected with miR‐140‐3p inhibitor, or NC inhibitor, or miR‐140‐3p inhibitor + siNC, or miR‐140‐3p inhibitor + siBCL9, or miR‐140‐3p inhibitor + siBCL2. Transfection was performed with Lipofectamine 2000 reagent (11668‐019, Invitrogen, USA) according to manufacturer's instruction.

### 2.5 Real‐time PCR assay

Total RNA rapid extraction kit (BioTeke, RP1201) was employed to extract total RNA from CRC cells or tissues. First strand of cDNA was synthesized using M‐MLV reverse transcriptase (Takara, Tokyo, Japan). Real‐time PCR analyses were performed by using SYBR Green (BioTeke, Beijing, China) and Taq HS Perfect Mix (Takara, Tokyo, Japan) on Exicycler™ 96 (Bioneer). The 2^−∆∆Ct^ method was applied to quantify fold change. The primer sequences were listed as follows: hsa‐miR‐140‐3p: RT‐primer: 5’‐GTTGGCTCTGGTGCAGGGTCCGAGGTATTCGCACCAGAGCCAACCCGTGG‐3′, forward: 5′‐TACCACAGGGTAGAACCACGG‐3′, reverse: 5′‐GCAGGGTCCGAGGTATTC‐3′; U6: RT‐primer: 5′‐GTTGGCTCTGGTGCAGGGTCCGAGGTATTCGCACCAGAGCCAACAAAATATGG‐3′, forward: 5′‐GCTTCGGCAGCACATATACT‐3′, reverse: 5′‐GTGCAGGGTCCGAGGTATTC‐3′; BCL2: forward: 5′‐CTGGGAGAACAGGGTACGATAA‐3′, reverse: 5′‐CTGGGAGAACAGGGTACGATAA‐3′; BCL9: forward: 5′‐CCAATCAGGGTAAACAGGG‐3′, reverse: 5′‐ AGGAGTCGGCGGAAATAC‐3′; GAPDH: forward: 5′‐GACCTGACCTGCCGTCTAG‐3′, reverse: 5′‐AGGAGTGGGTGTCGCTGT‐3′.

### 2.6 Cell Counting Kit‐8 (CCK‐8) assay

After transfection, 3 × 10^3^ cells were seeded into per well of 96‐well plates, and incubated at 37°C with 5% CO_2_. At different time points (24, 48 or 72 hours), 10 μl of CCK‐8 reagent (96992, Sigma, USA) was added to per well, and incubated for another 1 hour. The absorbance at 450 nm was then measured at designated times.

### 2.7 Cell migration assay

Transwell migration assay was performed with transwell chambers (3422, Corning USA). 5 × 10^3^ cells were seeded into the upper chambers of Transwell devices in 200 μl of serum‐free medium. The lower wells were added 800 μl complete medium with 30% of FBS. After 24 hours incubation, the migratory cells were fixed with 4% of paraformaldehyde (P6148, Sigma, USA) for 15 minutes, and then stained with 0.4% of crystal violet (0528, Amresco, USA) for 5 minutes. The images were taken with a microscope (DP73, Olympus, Japan) at 200× magnification.

### 2.8 Cell invasion assay

In cell invasion assay, transwell chambers were coated with a mixture of Matrigel (BD Biosciences, 356234) and serum‐free medium (1:3) prior to the assay. The subsequent steps were done as mentioned in cell migration assay section.

### 2.9 Immunofluorescence assay

Cells grown on coverslips were fixed with 4% of paraformaldehyde for 15 minutes, and permeabilized with 0.1% of Triton X‐100 (ST795, Beyotime, China) for 30 minutes. After blocking with goat serum, the sections were incubated with primary antibody against Vimentin (1:50; A19607, ABclonal Biotechnology, China) or β‐catenin (1:50; A19657, ABclonal Biotechnology, China) overnight at 4°C. After PBS washing, the sections were incubated with Cy3‐labeled secondary antibodies (1:200) at room temperature for 1 hour. Nuclei were stained with DAPI (D106471, Aladdin, China). The sections were visualized with a microscope at 400× magnification.

### 2.10 Protein extraction

To extract total protein, RIPA lysis buffer (R0010, Solarbio, China) were employed to lyse cells or tumor tissues.

For cytoplasmic and nuclear protein extraction, cells were lysed on ice with 200 μl of cytoplasmic lysis buffer. After 10 minutes, the samples were centrifuged (12,000× *g*, 10 minutes). The supernatant containing cytoplasmic protein was collected. The pellet was lysed with 50 μl of nuclei lysis buffer. After centrifugation, the obtained supernatant was nuclear protein.

### 2.11 Western blot analysis

The protein concentration was determined with BCA Protein Assay Kit (P0009, Beyotime, China). The protein extracts were separated in SDS‐PAGE and transferred to PVDF membranes. The membranes were blocked with 5% of nonfat dry milk for 1 hour and incubated with primary antibodies. After incubation at 4°C overnight, the membrane was incubated with corresponding secondary antibodies conjugated with HRP‐labeled IgG for 1 hour. Protein bands were visualized with enhanced chemiluminescence reagent (ECL) and analyzed using Gel‐Pro‐Analyzer software. GAPDH and Histone H3 were used as the internal control. Primary antibodies used were as follows: E‐cadherin antibody (ABclonal Biotechnology, A3044), N‐cadherin antibody (ABclonal Biotechnology, A19083), Vimentin antibody (ABclonal Biotechnology, A19607), Snail antibody (ABclonal Biotechnology, A5243), ZEB1 antibody (ABclonal Biotechnology, A5600), Bax antibody (ABclonal Biotechnology, A19684), cleaved‐caspase‐3 antibody (Affinity, AF7022), Bcl‐xL antibody (ABclonal Biotechnology, A19703), β‐catenin antibody (ABclonal Biotechnology, A19657), BCL9 antibody (ABclonal Biotechnology, A6795), BCL2 antibody (ABclonal Biotechnology, A0208), GAPDH antibody (Proteintech, 60004–1‐Ig), and Histone H3 antibody (Gene Tex, GTX122148).

### 2.12 Apoptosis assay

Annexin V‐FITC apoptosis detection kit (Beyotime, C1062) was employed to perform apoptosis assay. After 48 hours of transfection, cells were collected and resuspended with PBS. After centrifugation, cells were resuspended with 195 μl of Annexin V‐FITC binding buffer, and stained with 5 μl of Annexin V‐FITC and 10 μl of propidium iodide. The sample was incubated at room temperature in dark for 10–20 minutes, and then, detected using flow cytometry (Aceabio, NovoCyte).

### 2.13 Hoechst staining

Cells were seeded on chamber slides placed in the 12‐well plates and cultured for 24 hours. After transfection for 48 hours, cells were fixed with 4% of paraformaldehyde. After washing with PBS, cells were stained with Hoechst 33258 staining solution (Beyotime, C0003). The fluorescent images were captured with a fluorescence microscope at 400× magnification. The nucleus of live cells is normally blue, while condensed or fragmented and bright nuclei are observed in apoptotic cells.

### 2.14 miRNA target prediction

Online target‐gene prediction tools including TargetScan (http://www.targetscan.org), miRDB (http://mirdb.org), and RNAhybrid (https://bibiserv.cebitec.uni‐bielefeld.de/rnahybrid) were employed to predict target genes of miR‐140‐3p. Complementary seed match between miR‐140‐3p and the 3’UTR of target gene was also forecast with the above three prediction algorithms.

### 2.15 Luciferase reporter assay

Wild type (WT) and mutant type (MT) 3’UTR of BCL9 or BCL2 was cloned into pmirGLO vector, respectively. The constructed plasmids and miR‐140‐3p mimic and NC mimic were co‐transfected into 293T cells or LoVo cells. Firefly and Renilla luciferase activities were detected by dual‐luciferase assays (E1910, Promega, USA), and then, normalized to Renilla luciferase activity.

### 2.16 Lentivirus transduction

4 × 10^5^ LoVo cells were seeded into per well of 6‐well plates. After 24 hours, cells were transduced with the lentivirus expressing pre‐miR‐140 or the negative control for 48 hours (The MOI value of cells was 10). To obtain stable miR‐140 overexpression cells, the cells were incubated in the presence with G418 (400 μg/ml) for 1 week.

### 2.17 Tumor xenograft and metastasis model

Animal experiments were conducted according to Guide for the Care and Use of Laboratory Animals, and were approved by the Ethics Committee of Shengjing Hospital of China Medical University. Male nude mice (Balb/c) aged 5–6 weeks were randomly divided into four groups (each with 6 mice). For xenograft tumor formation, the mice were inoculated with LoVo‐NC or LoVo‐pre‐miR‐140 cells at 1 × 10^6^ into their subcutaneous flank. All tumor volumes were monitored every 5 days, and calculated as V = (length × width^2^)/2. At day 35 after the inoculation, mice were sacrificed, and tumors were photographed, fixed, and stored. For the metastasis assays, the mice (each with six mice) were anesthetized and fixed in supine position. The spleen was exposed by making a 1 cm incision in the left upper abdomen, and 1 × 10^6^ LoVo‐NC or LoVo‐pre‐miR‐140 cells were inoculated into the distal tip of the spleen. After inoculation, the incision was closed. At 35 days postinjection, mice were sacrificed, and the liver were dissected out, photographed, and fixed.

### 2.18 TUNEL staining

The sections of tumor tissues were dewaxed with xylene. Then, the sections were rehydrated in ethanol. Next, the sections were permeabilized using 0.1% of Triton X‐100 in citrate buffer for 8 min, then blocked with 3% of hydrogen peroxide for 10 minutes. The sections were incubated with TUNEL reaction mixture (enzyme solution: label solution = 1: 9) for 60 minutes without light. After incubation with converter‐POD solution for 30 minutes, the sections were subjected to color development with diaminobenzidine (DAB), and stained with hematoxylin. After differentiated in 1% of hydrochloric acid alcohol, the sections were dehydrated in ethanol and treated with xylene. The apoptotic cells were examined under an OLUMPUS microscope at 400× magnification.

### 2.19 Hematoxylin and eosin (HE) staining

The sections of liver tissues were dewaxed and rehydrated. After washing in Milli‐Q water, the sections were stained with hematoxylin (H8070, Solarbio, China), followed by staining with eosin (A600190, Sangon, China). The sections were then subjected to ethanol and xylene. The mounted sections were observed using a microscope at 200× magnification. Quantitative analysis of metastatic liver nodules was performed.

### 2.20 Statistical analysis

Statistical analysis was performed by using GraphPad Prism. All data were subjected to two‐tailed unpaired *t* test, paired *t* test, repeated measures ANOVA, or one‐way ANOVA followed by post hoc Tukey's test, wherever appropriate. The Pearson correlation analysis was used to measure correlation between the expression levels of miR‐140‐3p and BCL9 or BCL2 in CRC tissues. Statistical significance was inferred at *p* < 0.05 (*p < 0.05, **p < 0.01, ***p < 0.001, ****p < 0.0001).

## 3 RESULTS

### 3.1 miR‐140‐3p was downregulated in clinical CRC samples

**FIGURE 1:**
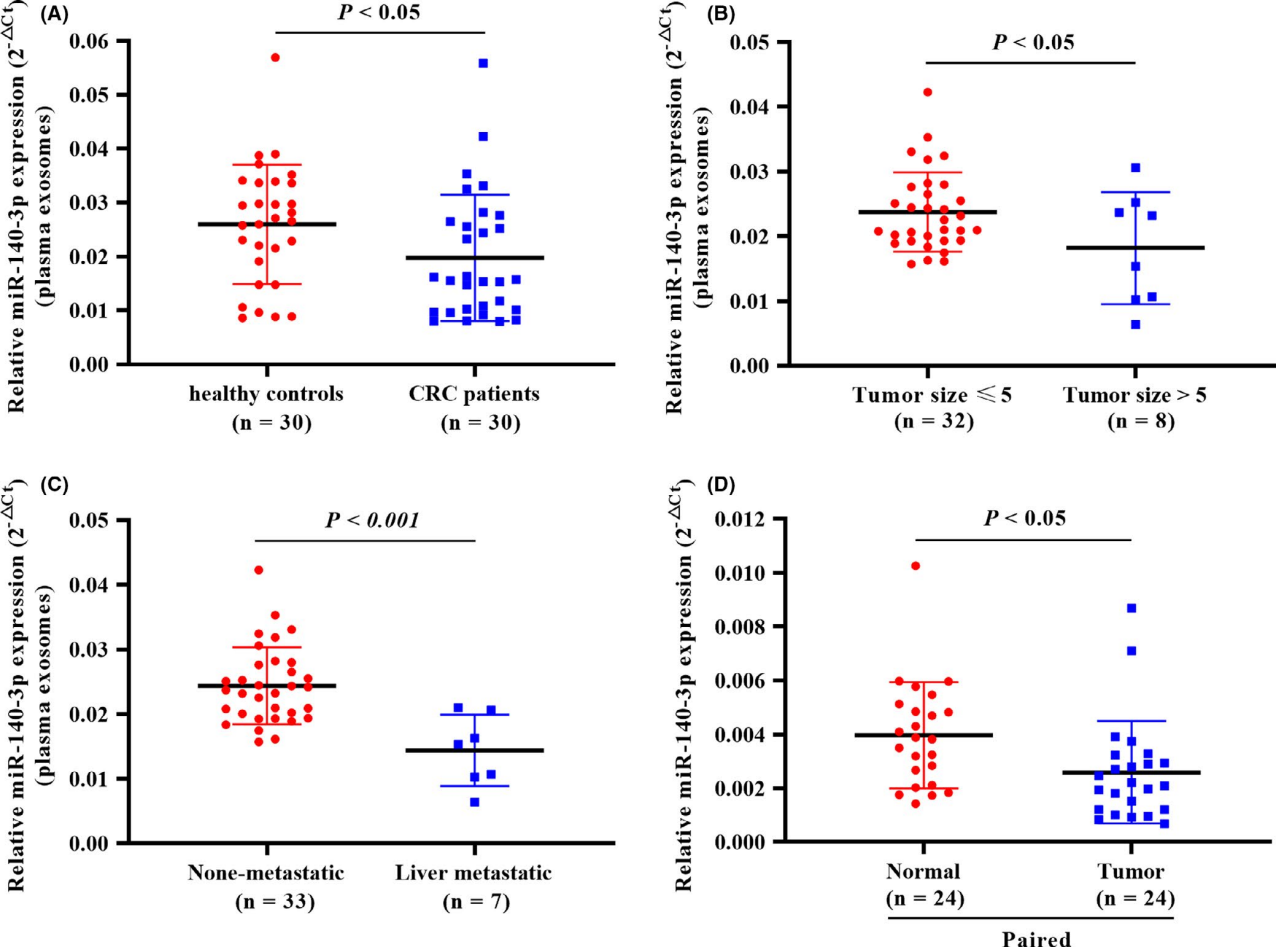
miR‐140‐3p was downregulated in clinical CRC samples. (A) The expression of miR‐140‐3p in plasma exosomes of healthy subjects (n = 30) and CRC patients (n = 30). (B) The level of miR‐140‐3p in plasma exosomes of CRC patients whose tumor size ≤5 cm (n = 32) and whose tumor size >5 cm (n = 8). (C) The level of miR‐140‐3p in plasma exosomes of CRC patients without metastasis (n = 33) and with liver metastasis (n = 7). (D) Real‐time PCR analyses of miR‐140‐3p in 24 cases containing normal and colorectal carcinoma tissues. Data are presented as relative expression levels. Two‐tailed unpaired *t*‐test for A–C, and paired *t* test for D

We first determined the expression of miR‐140‐3p in plasma exosomes of healthy donors (n = 30) and CRC patients (n = 30). Real‐time PCR analysis indicated that miR‐140‐3p from plasma exosomes was downregulated in CRC patients in comparison with healthy controls (Figure 1A). We further examined the level of miR‐140‐3p in plasma exosomes from a cohort of CRC patients (n = 40). The expression of miR‐140‐3p was prominently lower in CRC patients with tumor size >5 cm than that in CRC patients with tumor size ≤5 cm (Figure 1B). Moreover, decreased plasma exosomal miR‐140‐3p expression in liver metastatic patients was observed compared with none‐metastatic patients (Figure 1C). In addition, we collected tumor tissues and adjacent normal tissues from 24 CRC patients. Real‐time PCR analyses showed that miR‐140‐3p was decreased in tumor tissues in comparison with the corresponding normal tissues (Figure 1D).

### 3.2 Overexpression of miR‐140‐3p in CRC cells inhibited proliferation, migration, and invasion, and promoted apoptosis

**FIGURE 2:**
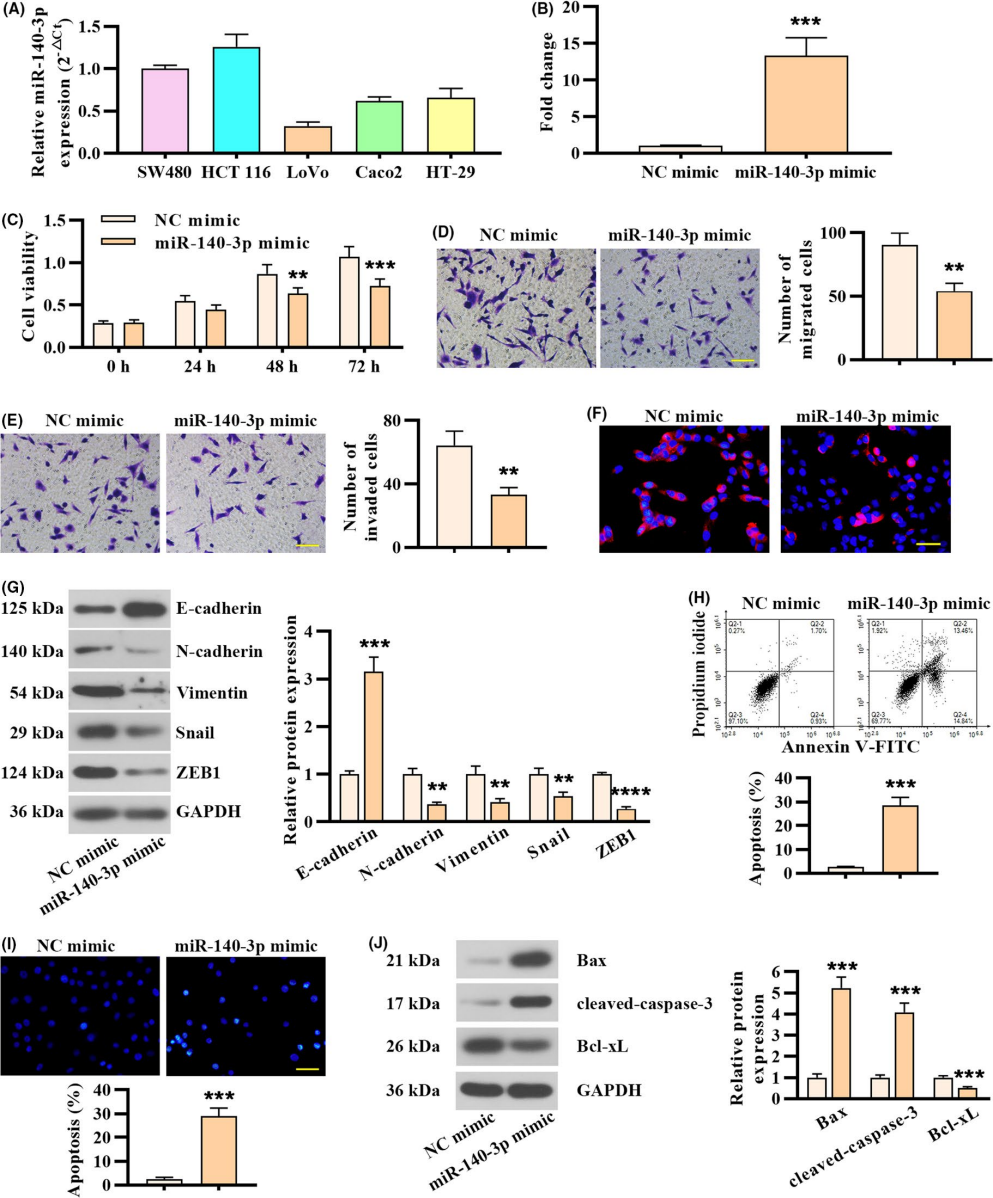
Overexpression of miR‐140‐3p in CRC cells inhibited proliferation, migration, and invasion, and promoted apoptosis. (A) miR‐140‐3p expression in CRC cell lines determined by real‐time PCR analysis. (B) miR‐140‐3p expression in LoVo cells following miR‐140‐3p mimic or negative control transfection. (C) Cell viability was tested at designated times. (D) Cell migration assay in LoVo cells following miR‐140‐3p overexpression. Scale bar, 100 μm. (E) Cell invasion assay in LoVo cells following miR‐140‐3p overexpression. Scale bar, 100 μm. (F) The expression of Vimentin detected by immunofluorescence staining. Scale bar, 50 μm. (G) Western blot analysis of EMT‐associated markers in LoVo cells following miR‐140‐3p overexpression. (H) Apoptosis was determined by FACS. (I) Hoechst staining was employed to determine apoptosis. Scale bar, 50 μm. (J) Western blot analysis was performed to examine the expression of apoptosis‐related markers. Data in B, D, E, G, H, I, and J were represented as mean ± SD (n = 3, two‐tailed unpaired *t* test), with repeated measures ANOVA for C

To study the role of miR‐140‐3p in CRC development, we first determined miR‐140‐3p expression in CRC cell lines (Figure 2A). We employed miR‐140‐3p mimic to overexpress miR‐140‐3p in LoVo cells showing its relatively lower expression. miR‐140‐3p overexpression efficiency was confirmed by real‐time PCR (Figure 2B). As shown in Figure 2C, miR‐140‐3p overexpression suppressed cell viability compared with that of NC mimic‐transfected cells (Figure 2C). Transwell assay elucidated that miR‐140‐3p overexpression decreased the migration and invasion ability of LoVo cells (Figure 2D,E). Giving that epithelial–mesenchymal transition (EMT) is activated during tumor invasion and metastasis, the immunofluorescence analysis of Vimentin (mesenchymal marker) was performed. The expression of Vimentin in cells transfected with miR‐140‐3p mimic was decreased in contrast to controls (Figure 2F). The results of Western blot confirmed that the protein level of Vimentin, N‐cadherin, Snail, and ZEB1 was downregulated in miR‐140‐3p overexpressed‐LoVo cells, and E‐cadherin protein was increased (Figure 2G). Furthermore, apoptosis assay was conducted by FACS and Hoechst staining. The results showed overexpression of miR‐140‐3p led to markedly higher apoptotic percentage (Figure 2H,I). Western blot analysis showed overexpression of miR‐140‐3p resulted in upregulation of Bax and cleaved‐caspase‐3, but downregulation of Bcl‐xL (Figure 2J). Hence, miR‐140‐3p suppressed proliferation, migration, and invasion, and promoted apoptosis in CRC cells.

### 3.3 Inhibition of miR‐140‐3p in CRC cells promoted proliferation, migration, and invasion, and suppressed apoptosis

**FIGURE 3:**
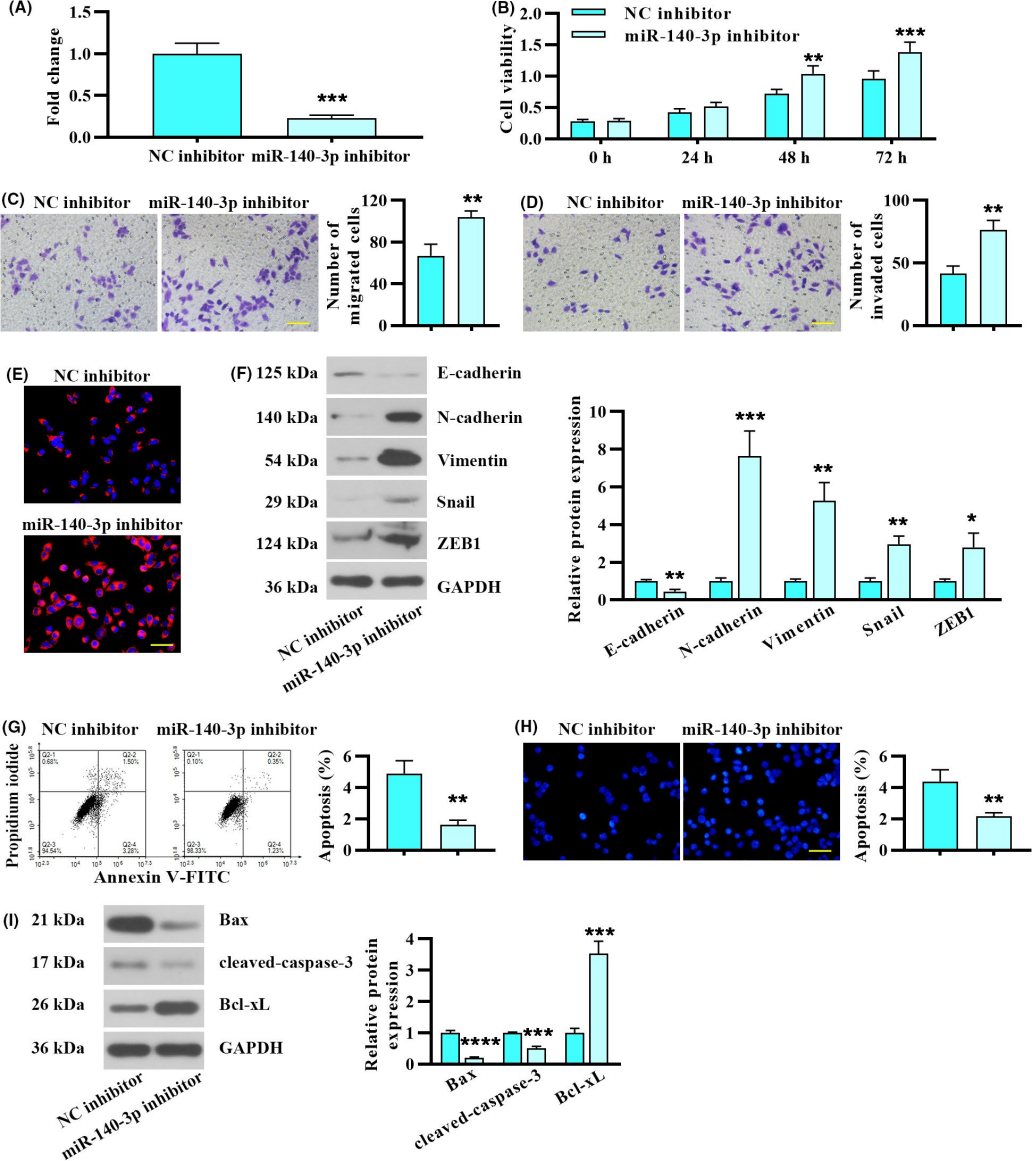
Inhibition of miR‐140‐3p in CRC cells promoted proliferation, migration, and invasion, and suppressed apoptosis. HCT 116 cells were transfected with miR‐140‐3p inhibitor or negative control. (A) miR‐140‐3p expression in HCT 116 cells following miR‐140‐3p inhibitor or negative control transfection. (B) Cell viability. (C) Transwell migration assay. Scale bar, 100 μm. (D) Effects of miR‐140‐3p silencing on cell invasion. Scale bar, 100 μm. (E) Vimentin expression was analyzed by immunofluorescence staining. Scale bar, 50 μm. (F) Inhibition of miR‐140‐3p affected the expression of EMT biomarkers. (G) Apoptosis of HCT 116 cells was determined by FACS. (H) Apoptosis was evaluated by Hoechst staining. Scale bar, 50 μm. (I) The levels of apoptosis‐related markers following miR‐140‐3p inhibition. Data in A, C, D, F, G, H, and I were present by mean ± SD (n = 3, two‐tailed unpaired *t* test), with repeated measures ANOVA for panel B

Next, HCT 116 cells, which displayed relatively high miR‐140‐3p levels, were subjected to miR‐140‐3p silence with its inhibitor. The transfection efficiency was validated and presented in Figure 3A. miR‐140‐3p inhibitor‐transfected HCT 116 cells exhibited increased cell viability in contrast to NC inhibitor‐transfected cells (Figure 3B). miR‐140‐3p knockdown in HCT 116 cells resulted in increased cell migration and invasion capacities (Figure 3C,D). Both immunofluorescence analysis of Vimentin and Western blot analysis indicated miR‐140‐3p knockdown promoted EMT progression in CRC cells (Figure 3E,F). Moreover, the results of FACS and Hoechst staining showed that miR‐140‐3p knockdown suppressed apoptosis of HCT 116 cells (Figure 3G,H), which was proved by decreased expression of Bax and cleaved‐caspase‐3, but upregulation of Bcl‐xL (Figure 3I). These results together suggested that silence of miR‐140‐3p in HCT 116 cells promoted proliferation, migration, and invasion, and suppressed apoptosis.

### 3.4 miR‐140‐3p affected β‐catenin nuclear localization in CRC cells

**FIGURE 4:**
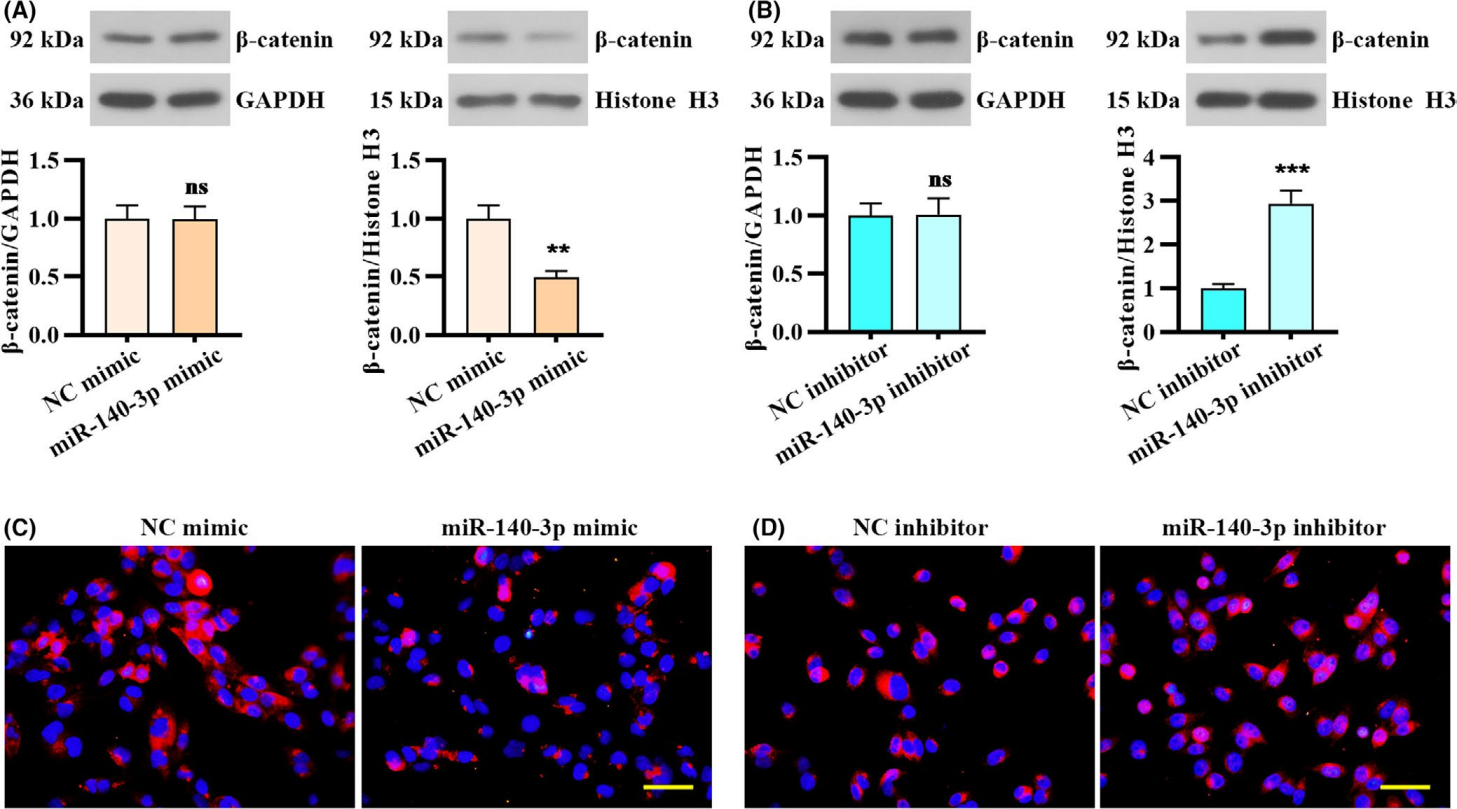
miR‐140‐3p affected β‐catenin nuclear localization in CRC cells. (A and B) Western blot analysis showed the expression of nuclear and cytoplasmic β‐catenin in CRC cells following miR‐140‐3p overexpression or inhibition. (C and D) Immunofluorescence showed subcellular localization of β‐catenin. Scale bar, 50 μm. Data were presented by mean ± SD with two‐tailed unpaired *t* test

Considering Wnt/β‐catenin signaling pathway is implicated in EMT and cancer cell metastasis,^29^, ^30^ we further investigated β‐catenin was modulated by miR‐140‐3p. Western blot analysis indicated that miR‐140‐3p overexpression decreased the protein levels of nuclear β‐catenin, and did not affect cytoplasmic β‐catenin expression (Figure 4A). In contrast, the level of nuclear β‐catenin was increased in HCT 116 cells transfected with miR‐140‐3p inhibitor (Figure 4B). Similarly, immunofluorescence staining showed that miR‐140‐3p overexpression suppressed nuclear translocation of β‐catenin in LoVo cells (Figure 4C). However, following miR‐140‐3p knockdown, β‐catenin nuclear translocation was triggered in HCT 116 cells (Figure 4D). Above results revealed that the nuclear translocation of β‐catenin may be modulated by miR‐140‐3p.

### 3.5 miR‐140‐3p‐targeted 3’UTR of BCL9 and BCL2, and modulated their expression

**FIGURE 5:**
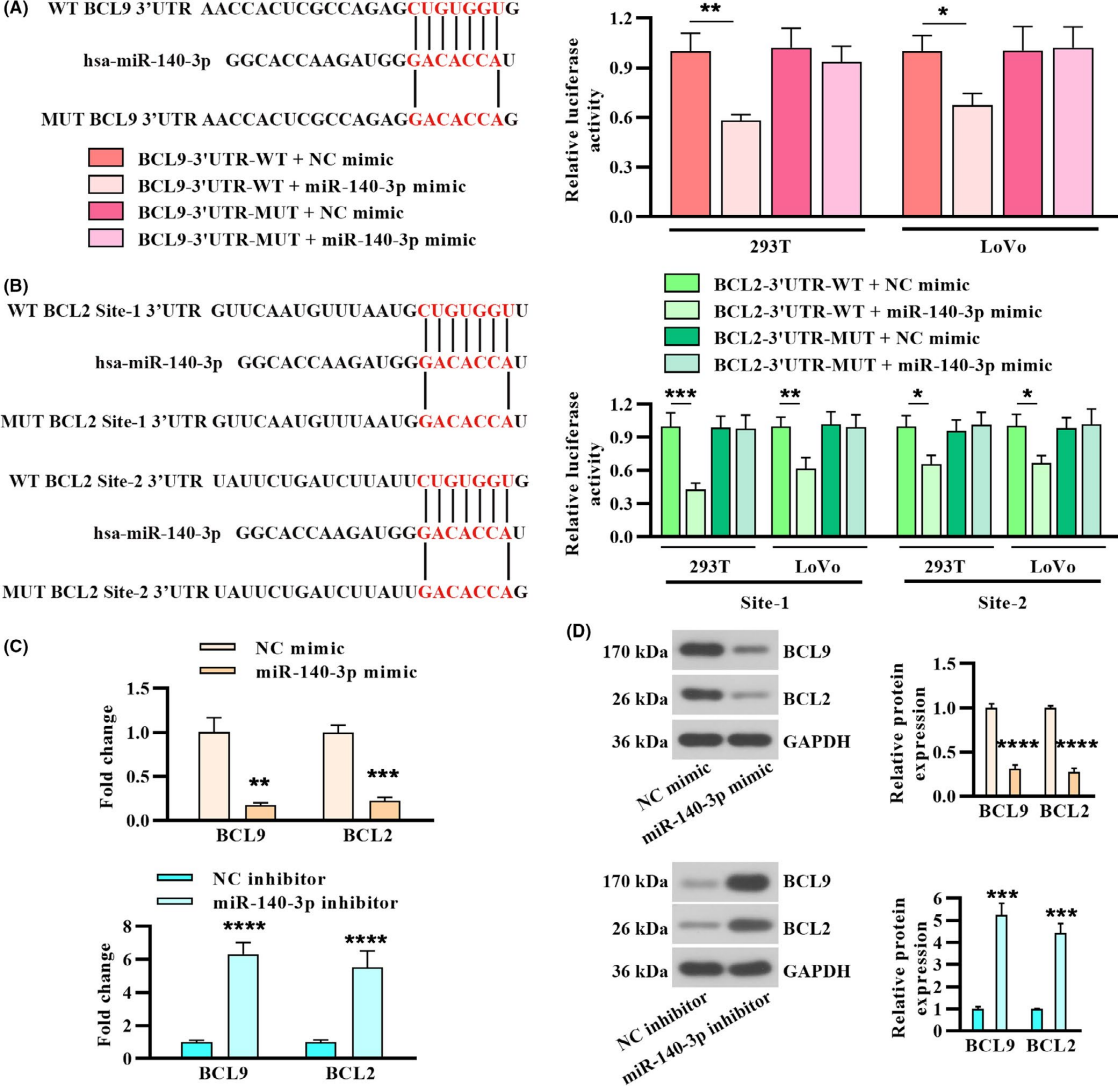
miR‐140‐3p‐targeted 3′UTR of BCL9 and BCL2, and modulated their expression. (A) Left panel: prediction of miR‐140‐3p target site on human BCL9 3’UTR (NM_004326). Right panel: binding of miR‐140‐3p and BCL9 validated by luciferase assay of the indicated cells. (B) Two predicted miR‐140‐3p‐binding site on 3’UTR of human BCL2 (NM_000633) and relative luciferase activity. (C) Real‐time PCR analysis of BCL9 and BCL2 in CRC cells following miR‐140‐3p overexpression or inhibition. (D) The protein levels of BCL9 and BCL2 and quantitative analysis in CRC cells. Data were represented by mean ± SD (n = 3)

To explore the molecular mechanism of miR‐140‐3p in suppressing CRC development, the potential targets of miR‐140‐3p were searched by bioinformatics prediction. BCL9 was found to be a potential novel target of miR‐140‐3p. Moreover, BCL2 is a known target of miR‐140‐3p.^31^ Complementary seed match between miR‐140‐3p and the 3’UTR of BCL9 or BCL2 forecasted by prediction algorithms is shown in Figure 5A,B. To validate the predicted miRNA‐binding site, the BCL9 or BCL2 3’UTR and their mutated 3’UTR constructs were generated (Figure 5A,B). Then, 293T cells and LoVo cells were co‐transfected with the constructs and miR‐140‐3p mimic. Relative luciferase activity of the wild‐type reporter was repressed by miR‐140‐3p overexpression, while the mutant reporter was not affected (Figure 5A,B). In addition, miR‐140‐3p overexpression markedly downregulated BCL9 and BCL2 mRNA levels in LoVo cells, while its inhibition upregulated BCL9 and BCL2 mRNA levels in HCT 116 cells (Figure 5C). miR‐140‐3p overexpression or inhibition triggered changes in BCL9 and BCL2 protein expression were consistent with mRNA levels (Figure 5D). These results proved that miR‐140‐3p‐targeted 3’UTR of BCL9 and BCL2, and modulated their expression.

### 3.6 miR‐140‐3p affected proliferation, migration, invasion, and apoptosis in CRC cells by regulating BCL9 and BCL2

**FIGURE 6:**
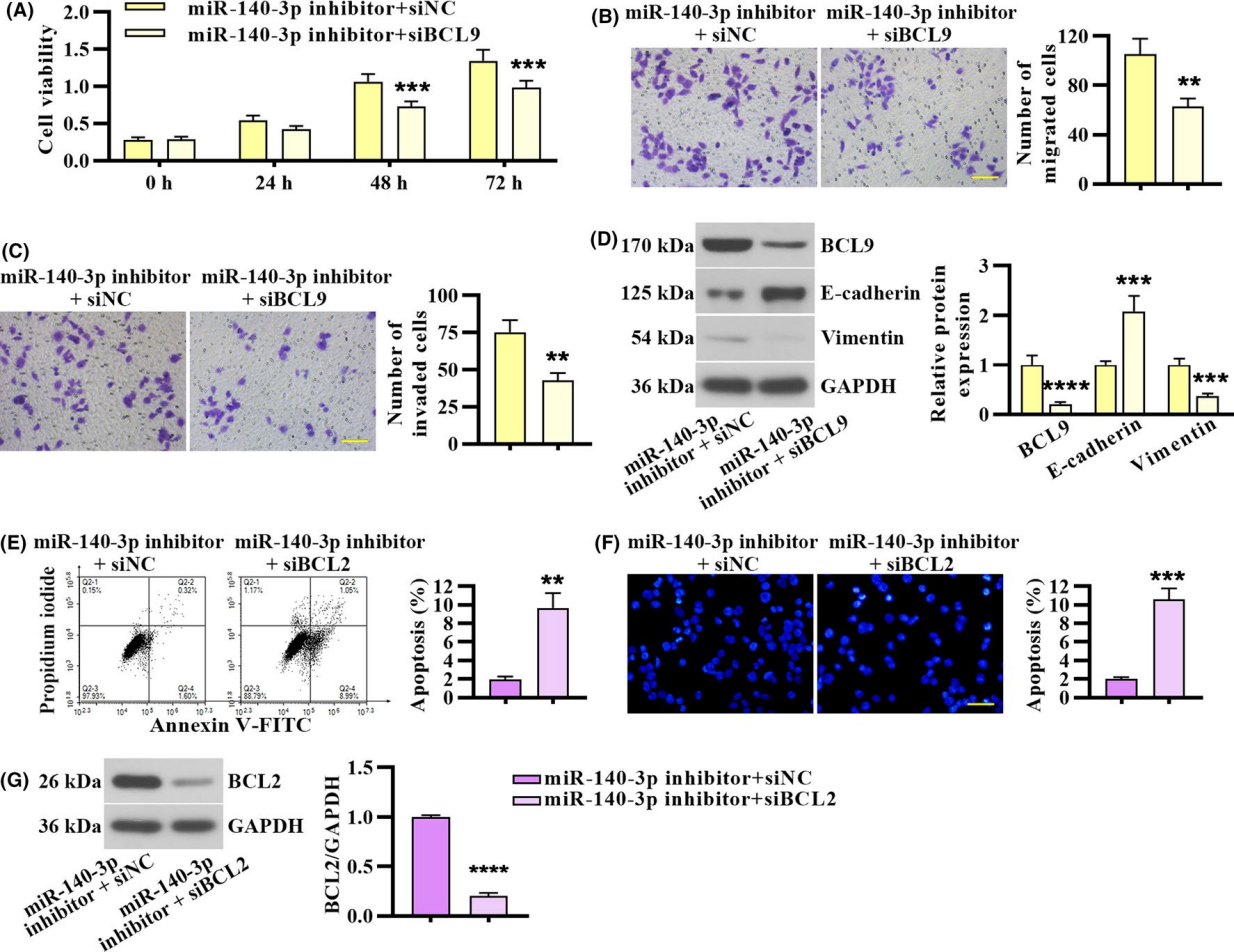
miR‐140‐3p affected proliferation, migration, invasion, and apoptosis in CRC cells by regulating BCL9 and BCL2. miR‐140‐3p silenced HCT 116 cells were co‐transfected with BCL9 siRNA or siNC. (A) Cell viability. (B & C) Transwell migration and invasion assay. Scale bar, 100 μm. (D) The protein level of BCL9, E‐cadherin, and Vimentin. miR‐140‐3p silenced HCT 116 cells were co‐transfected with BCL2 siRNA or siNC. (E) Apoptosis assay was performed by FACS. (F) Apoptosis was evaluated by Hoechst staining. Scale bar, 50 μm. (G) BCL2 expression in HCT 116 cells co‐transfected with miR‐140‐3p inhibitor and BCL2 siRNA. Data in panel A were presented by mean ± SD with repeated measures ANOVA (n = 3). Data in B–G were calculated from two‐tailed unpaired *t* test (n = 3)

To investigate whether knockdown of BCL9 and BCL2 counteracts the effects of miR‐140‐3p inhibition on CRC cells, HCT 116 cells were transfected with miR‐140‐3p inhibitor and siRNA of BCL9 or BCL2. Knockdown of BCL9 suppressed proliferation, migration, and invasion capacities in miR‐140‐3p silenced HCT 116 cells (Figure 6A–D). Furthermore, knockdown of BCL2 induced apoptosis in miR‐140‐3p silenced HCT 116 cells (Figure 6E–G). These results validated that miR‐140‐3p affected proliferation, migration, invasion, and apoptosis by regulating BCL9 and BCL2 in CRC cells.

### 3.7 BCL9 and BCL2 were upregulated in CRC tissues and inversely correlated with miR‐140‐3p expression

**FIGURE 7:**
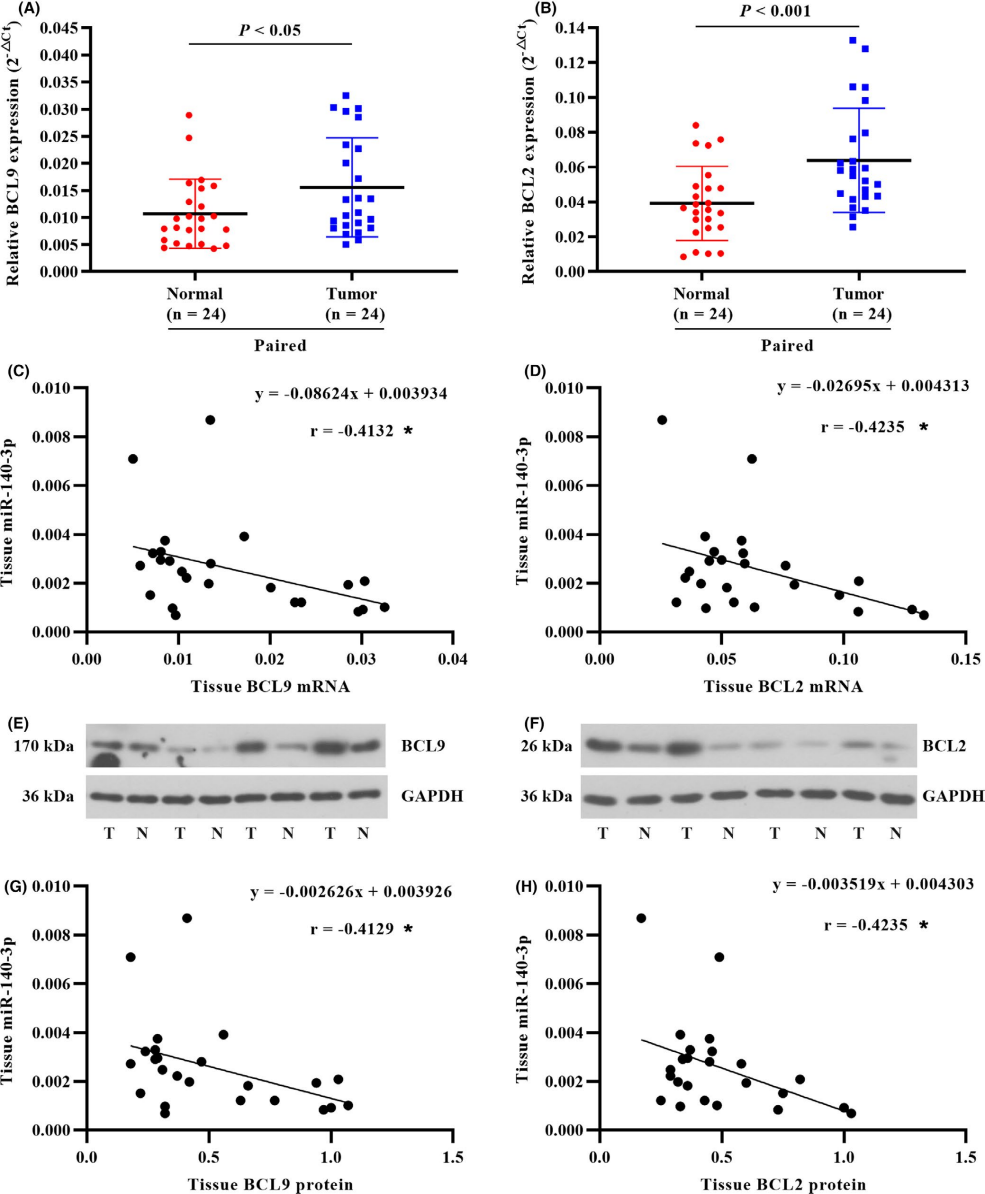
BCL9 and BCL2 were upregulated in CRC tissues and inversely correlated with miR‐140‐3p expression. (A and B) BCL9 and BCL2 mRNA levels were determined in the paired CRC tissues. (C and D) Correlation graph for tumor tissue miR‐140‐3p and BCL9 or BCL2 mRNA. Pearson's correlation coefficient (r) and *P* value (*p < 0.05) were presented. (E and F) The representative blots of BCL9 and BCL2 protein expression in the normal tissue (N) and tumor tissue (T). (G and H) Correlation graph for tumor tissue miR‐140‐3p and BCL9 or BCL2 protein. Data in A & B were calculated from paired *t*‐test. Data in C, D, G, and H were determined by Pearson correlation analysis

Similarly, we determined the expression of BCL9 and BCL2 in 24 paired CRC tissues. The mRNA levels of BCL9 and BCL2 were found to be upregulated in the tumor tissues compared with adjacent normal tissues (Figure 7A,B). Furthermore, miR‐140‐3p expression level and BCL9 or BCL2 mRNA level in tumor tissues were negatively correlated (Figure 7C,D). In addition, BCL9 and BCL2 protein levels were increased in tumor tissues compared with adjacent normal tissues (Figure 7E,F). The results in Figure 7G,H indicated miR‐140‐3p expression level was inversely correlated with BCL9 or BCL2 protein level.

### 3.8 miR‐140‐3p suppressed tumor growth and liver metastasis in LoVo cell xenograft mouse model

**FIGURE 8:**
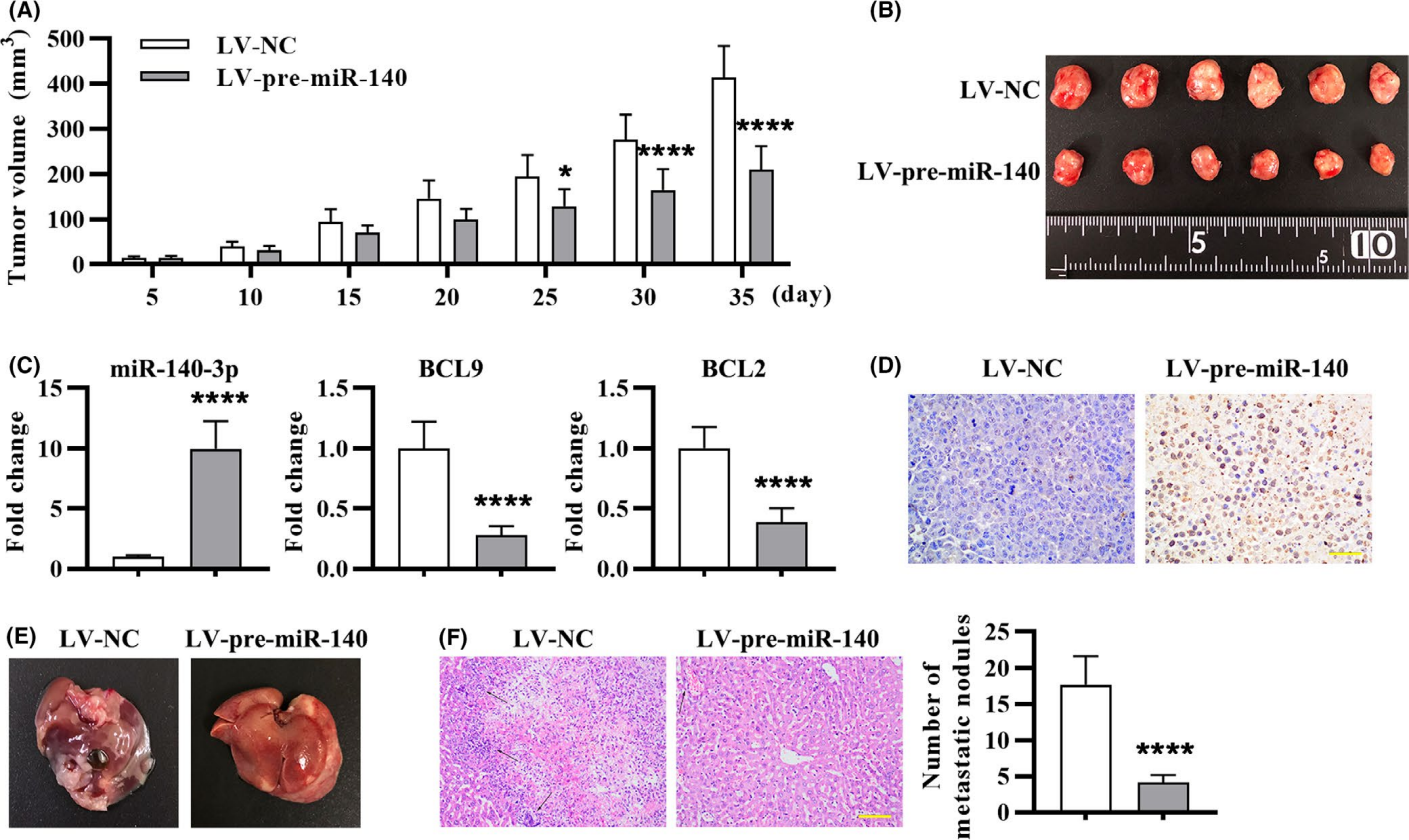
miR‐140‐3p suppressed tumor growth and liver metastasis in LoVo cell xenograft mouse model. The nude mice were subjected to subcutaneous injection of LoVo cells: (A) Tumor volume was monitored from days 5 to day 35. (B) The images of tumor at 35 days after subcutaneous injection. (C) miR‐140‐3p, BCL9, and BCL2 expression in tumor tissues was detected by real‐time PCR. (D) TUNEL staining of tumor tissue sections to detect apoptosis. Thirty‐five days after tail vein injection of LoVo cells: (E) The liver morphology and nodules were presented. (F) HE staining of metastatic liver tissues and quantitation of (F) by counting hepatic metastatic nodules. The metastatic nodules were indicated with black arrows. Data were presented by mean ± SD (n = 6), with repeated measures ANOVA for A, and two‐tailed unpaired *t* test for C and F

We further determined whether miR‐140‐3p exerted as a tumor suppressor in vivo. miR‐140‐3p‐overexpressed‐LoVo cells (LV‐pre‐miR‐140) or control cells (LV‐NC) were inoculated subcutaneously in nude mice. Compared with the control group, tumor volume was decreased in LV‐pre‐miR‐140 group (Figure 8A,B). To confirm whether miR‐140‐3p overexpression regulates the expression of BCL9 and BCL2, we performed real‐time PCR on xenograft tumor tissues. The results indicated that overexpression of miR‐140‐3p downregulated the levels of BCL9 and BCL2 (Figure 8C). TUNEL staining of xenograft tumor sections indicated apoptosis in LV‐pre‐miR‐140 group was increased (Figure 8D). To investigate the role of miR‐140‐3p in liver metastasis, we injected LoVo cells into the distal tip of the spleen. After 35 days, mice were sacrificed and the livers were collected. Overexpression of miR‐140 led to fewer metastatic nodules (Figure 8E). Besides, the results of the HE staining showed that tumor cells were in irregular shape of control mice, and the nuclei were swelling, abnormal, and hyperchromatic. However, the density of tumor cells and the number of the irregular cells in LV‐pre‐miR‐140 group was reduced. Moreover, the number of hepatic metastatic nodules in LV‐pre‐miR‐140 mice observed by microscope was also reduced compared with the LV‐NC group (Figure 8F). Hence, miR‐140‐3p suppressed tumor growth and liver metastasis in mouse model.

## 4 DISCUSSION

In the current study, low miR‐140‐3p expression was detected in the plasma exosomes and tumor tissues of CRC. Moreover, low expression of miR‐140‐3p was correlated with CRC liver metastasis. Based on these results, we concluded that miR‐140‐3p may inhibit the aggressive tumor phenotype in CRC. Since the functions of miRNA depend on its targets, we predicted and confirmed BCL9 and BCL2 as direct targets of miR‐140‐3p. miR‐140‐3p suppressed proliferation, migration, invasion, and β‐catenin nuclear localization in CRC cells by regulating BCL9, and BCL2 was involved in miR‐140‐3p‐mediated apoptosis. In vivo study confirmed that miR‐140‐3p suppressed CRC tumor growth and metastasis. These findings collectively suggested that targeting miR‐140‐3p‐BCL9/BCL2 axis might be a potential treatment strategy for CRC.

miRNAs in plasma/serum are emerging as noninvasive biomarkers for cancer patients.^32^, ^33^ Previous studies have reported circulating miRNAs from plasma/serum as diagnostic biomarkers and prognostic factors in CRC. Du et al. validated plasma miR‐21 was a reliable biomarker for CRC diagnosis which had a relatively high diagnostic efficiency.^34^ Serum miR‐203 level of CRC patients was upregulated in a stage‐dependent manner, suggesting it could be a prognostic and predictive biomarker in CRC.^35^ It was illuminated that cell‐derived exosomes are the possible source of serum or plasma miRNAs.^36^ Under the protection of the bilayer membrane structure of exosomes, miRNAs in plasma/serum exosomes are segregated from endogenous RNase in blood, which are in a stable form. Therefore, exosomes containing intact miRNAs may be utilized for the detection and management of cancer.

In our study, we found low level of miR‐140‐3p in plasma exosomes were associated with CRC liver metastasis, suggesting the potential of plasma exosomal miR‐140‐3p as a biomarker for prediction of metastasis in CRC patients. As demonstrated in previous study, miR‐140‐3p also can exert as an independent predictor of cutaneous melanoma patient survival, because its strong expression is associated with favorable survival of cutaneous melanoma patients.^37^ Functional experiments in this study demonstrated that increasing intracellular miR‐140‐3p expression suppressed migration, invasion, and EMT process. Opposing results were observed when miR‐140‐3p was silenced. In vivo experiments showed miR‐140‐3p inhibited CRC liver metastasis, which further confirmed the above findings in CRC cells. One limitation of this study is that we preliminarily explored the role of miR‐140‐3p in CRC cells and mouse model. Future work needs to be done to investigate the role of CRC‐derived exosomal miR‐140‐3p, and further substantiate exosomal miR‐140‐3p as biomarkers for CRC patients.

Next, we explored the mechanism of miR‐140‐3p‐mediated tumor growth and metastasis in CRC. miRNA target prediction algorithm and luciferase assay confirmed that BCL9 is a target gene of miR‐140‐3p. Mani et al. has demonstrated that BCL9 is highly expressed in CRC tumor tissues and cell lines, and it promotes proliferation, migration, and invasion potential of CRC cells.^21^ They also corroborated that BCL9 promotes EMT, a progression implicated in the metastasis of tumors. The expression profiling data of BCL9 knockdown cells revealed that BCL9 not only regulates epithelial/mesenchymal markers, but also regulates a subset of genes involved in EMT.^21^ Moreover, BCL9, a co‐activator for β‐catenin‐modulated transcription, binds to the surface of β‐catenin through its α‐helical homology domain 2 and drives β‐catenin signaling.^38^ It was confirmed that disruption of the interaction between BCL9 and β‐catenin selectively inhibits oncogenic Wnt transcription, thereby suppressing tumor growth and metastasis in CRC.^25^ In the current study, we determined the level of BCL9 in normal and tumor tissues of CRC patients. As expected, BCL9 expression in tumor tissues was markedly higher than that in normal tissues of CRC patients. We also substantiated inhibition of miR‐140‐3p conferred increased proliferation, migration, invasion, and EMT properties to CRC cells, while knockdown BCL9 in miR‐140‐3p inhibitor‐transfected CRC cells blocked these enhancements. Therefore, miR‐140‐3p suppressed tumor growth and metastasis in CRC by directly targeting BCL9.

In this study, BCL2 was confirmed to be implicated in miR‐140‐3p‐modulated pro‐apoptosis effect in CRC cells. BCL2 is required for cancer cells to survive.^39^ Pro‐apoptotic factors are activated following cellular damage or deregulation. Some cancer cells that overexpress BCL2 can escape apoptosis by binding or sequestering pro‐apoptotic factors.^40^ Consistently, high expression of BCL2 was found in tumor tissues of CRC patients in contrast to normal tissues. Currently, small‐molecule inhibitors, that binds to pro‐survival BCL2 to actuate apoptosis of malignant cells, are applied in clinical therapy of cancer.^41^, ^42^ In the present study, BCL2 knockdown restored apoptosis in miR‐140‐3p inhibitor‐transfected CRC cells, which may propose miR‐140‐3p/BCL2 axis as a therapeutic target in CRC.

## 5 CONCLUSIONS

In conclusion, we indicated that miR‐140‐3p had a low expression in tumor tissues of CRC, as well as the expression of plasma exosomal miR‐140‐3p was decreased in CRC patients and was correlated with liver metastasis. Furthermore, we provided evidence for miR‐140‐3p as a tumor suppressor in CRC progression, regulated cell proliferation, migration, invasion, and apoptosis via targeting BCL9 and BCL2. Thus, miR‐140‐3p may have a potential application in CRC diagnostics and treatment.

## CONFLICT OF INTEREST

The authors declare no conflict of interest.

## AUTHOR CONTRIBUTIONS

The study was designed by HZ. The experiments were performed by DL and CC. The data were collected and analyzed by MC. The manuscript was written by DL and revised by HZ. The funding was obtained by HZ.

## Data Availability

All data generated or analyzed during this study are included in this published article.
